# Complex Diagnostic Dilemma of Viral Hepatitis vs. Drug-Induced Hepatitis: A Case Report

**DOI:** 10.7759/cureus.49191

**Published:** 2023-11-21

**Authors:** Nava R Sharma, Pranay Siriya, Samar Pal Singh Sandhu, Madalasa Pokhrel, Diana Ching

**Affiliations:** 1 Internal Medicine, Maimonides Medical Center, New York, USA; 2 Internal Medicine, Montefiore New Rochelle Hospital, New Rochelle, USA; 3 Gastroenterology, Maimonides Medical Center, New York, USA

**Keywords:** ebv serology, ebv, hepatic failure, cocaine induced liver injury, drug-induced liver injury (dili), viral hepatitis b

## Abstract

This case report explores the intricate diagnostic challenges encountered in a 30-year-old male patient with abdominal pain, jaundice, and a history of acetaminophen use. Initially presenting as a potential case of drug-induced hepatitis due to acetaminophen overdose, the diagnosis took an unexpected turn when the patient tested positive for hepatitis B surface antigen. The case highlights the complexity of diagnosing acute hepatitis, considering multiple potential etiologies, including viral and drug-induced factors. Differential diagnoses for this case involve considering drug-induced hepatitis, autoimmune hepatitis, various viral hepatitis types, and the potential contribution of cocaine-induced hepatitis as individual possibilities or in combination.

This case emphasizes the need for a comprehensive evaluation, the consideration of multiple potential causes, and the importance of ongoing monitoring and follow-up to ensure optimal patient care in cases of acute hepatitis.

## Introduction

Acetaminophen overdose has emerged as a significant contributor to overdose-related acute liver failure (ALF) and fatalities in the United States, constituting a substantial portion of ALF cases. It is noteworthy that in the United States, overdoses involving acetaminophen are the primary cause of calls made to Poison Control Centers, with an annual incidence exceeding 100,000 [1]. These overdoses lead to more than 56,000 emergency room visits, resulting in approximately 2,600 hospitalizations and, tragically, around 450 deaths attributed to ALF each year [1,2]. Such cases of ALF attributed to acetaminophen may stem from intentional overdoses or unintentional ingestions, often influenced by various factors, including the concurrent use of alcohol and specific medications known to promote the formation of reactive and harmful metabolites [3].

Simultaneously, hepatitis B presents a global public health challenge with diverse modes of transmission. It can manifest in various forms, further complicated by concurrent infections and clinical factors, making diagnosis a complex task. This case explores the presentation of a previously healthy 30-year-old male with symptoms initially suggestive of acetaminophen-induced hepatotoxicity. However, the subsequent discovery of a positive hepatitis B surface antigen (HBsAg) raised questions about the role of viral hepatitis in his condition.

## Case presentation

We present the case of a 30-year-old male with no significant past medical history who presented to the emergency department with a three-day history of persistent abdominal pain, nausea, and vomiting. He mentioned experiencing flu-like symptoms a week before seeking medical attention. During that period, the patient took oral acetaminophen regularly, around five to eight times a day, with each dose containing 500mg of acetaminophen. This regimen continued for five days. The patient did not measure his temperature but reported feeling feverish without experiencing chills or rigor. The abdominal pain, graded at 10/10 in severity, was diffuse and lasted for approximately one to two hours, exacerbated after meals but spontaneously relieved. Accompanying the pain were episodes of non-projectile, non-bilious vomiting, occurring postprandially and during transit to the hospital. No history of diarrhea, constipation, chest pain, or similar abdominal pain episodes in the past was reported. The patient denied any prior blood transfusions, recent travel, or sick contacts within his family. He acknowledged one female sexual partner and denied any symptoms in his partner. Although he admitted to occasional alcohol consumption and previous cocaine use via snorting, he denied any other illicit drug intake or herbal medications.

On further examination, the patient presented with pallor and icterus, indicating a potential underlying hepatic involvement. There were no signs of acute distress, and the patient appeared conscious and oriented. The respiratory examination revealed normal breath sounds without any adventitious sounds. Cardiovascular examination demonstrated a pulse rate of 60 beats per minute, with no murmurs or abnormal heart sounds. The blood pressure was 110/60 mmHg, and the patient exhibited adequate perfusion. The abdomen was soft but tender on palpation, especially in the right upper quadrant, with no palpable organomegaly. Bowel sounds were present and normal. Peripheral edema and clubbing were absent. The neurological examination revealed no focal deficits, and the patient was alert and cooperative throughout the evaluation.

Given the patient's history of consuming acetaminophen (paracetamol) at higher than recommended doses for five days and the presentation with abdominal pain, nausea, vomiting, and jaundice, a suspicion of paracetamol toxicity was raised. As a result, the patient was immediately started on N-acetylcysteine (NAC) therapy in the emergency room as per the gastroenterology team's recommendation. Supportive measures, such as intravenous fluids and antiemetics, were provided to alleviate symptoms and maintain hydration. Additionally, investigations were performed to rule out other potential causes of hepatitis, such as viral or autoimmune etiologies, considering the initial uncertainty between viral hepatitis and drug-induced hepatitis.

Initial laboratory reports revealed abnormal liver function tests, including significantly elevated levels of alanine aminotransferase at 1631 U/L and aspartate aminotransferase at 800 U/L, well above the normal range. Alkaline phosphatase and total bilirubin levels were also elevated. The initial lab value has been tabulated in Table 1. Furthermore, the patient tested positive for HBsAg, suggesting a concurrent hepatitis B infection. The international normalized ratio was slightly elevated, indicating potential liver dysfunction.

**Table 1:** Initial laboratory value

Lab test	Normal range	Patient's result
Hemoglobin (Hb)	13.5-17.5 g/dL	14 g/dL
White blood cells (WBC)	4.5-11.0 x 10^3/μL	8 x 10^3/μL
Platelet count (PLT)	150-450 x 10^3/μL	200 x 10^3/μL
Alanine aminotransferase (ALT)	5-40 U/L	1700 U/L
Aspartate aminotransferase (AST)	5-35 U/L	800 U/L
Alkaline phosphatase (ALP)	30-120 U/L	200 U/L
Total bilirubin (TBIL)	0.2-1.2 mg/dL	8 mg/dL
Direct bilirubin (DBIL)	0-0.3 mg/dL	4.9 mg/dL
Total protein	6.0-8.3 g/dL	7 g/dL
International normalized ratio (INR)	0.8-1.2	1.2
Blood urea nitrogen (BUN)	7-20 mg/dL	14 mg/dL
Serum creatinine (Cr)	0.6-1.2 mg/dL	0.8 mg/dL
Random blood glucose (RBS)	70-99 mg/dL	90 mg/dL
Sodium (Na)	135-145 mmol/L	136 mmol/L
Potassium (K)	3.5-5.0 mmol/L	4 mmol/L
Ethanol (alcohol) level	<10 mg/dL	<10 mg/dL
Salicylate level	<2.5 mg/dL	<2.5 mg/dL
Acetaminophen level	<10 mg/dL	<10 mg/dL

**Figure 1:**
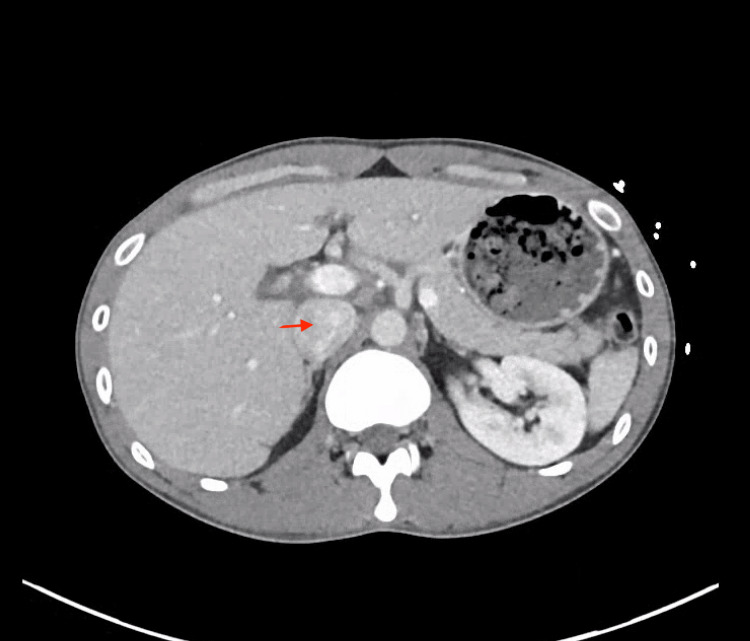
Prominent inferior vena cava as shown by a red arrow

A CT abdominal and pelvis contrast study showed the liver was normal in size and contour with homogeneous enhancement. There were no masses, but intrahepatic periportal edema was noted. The gallbladder appeared diffusely edematous without any biliary abnormality. It's worth noting that while the CT scan revealed a prominent inferior vena cava (IVC), as in Figure 1, the subsequent echocardiogram (ECHO) did not detect any evidence of right heart failure (RHF) or valvular pathology.

The patient was admitted for an acute hepatitis workup and management, which included additional tests to evaluate potential causes of hepatitis, such as hepatitis D, autoimmune markers, and viral infections like cytomegalovirus and varicella. All the markers have been tabulated in Table 2. Hepatitis B treatment was started with oral antiviral as per GI recommendation. The patient's history of drug use, including ketamine and cocaine, prompted a urine toxicology screen, which was negative. Cardiac monitoring was ongoing to address the bradycardia observed during the admission.

**Table 2:** Displaying various viral markers

Test	Patient's result
Hepatitis B surface antigen (HBsAg)	Positive
Hepatitis B surface antibody (HBsAb)	Non-reactive
Hepatitis B e antigen (HBeAg)	Non-reactive
Hepatitis B e antibody (HBeAb)	Positive
Hepatitis B core antibody (HBcAb)	Non-reactive
Hepatitis C antibody (anti-HCV)	Non-reactive
Hepatitis C RNA (HCV RNA)	Not Detected
Hepatitis D antibody (anti-HDV)	Non-reactive
Hepatitis E IgM antibody (HEV IgM)	Non-reactive
Herpes simplex 1 IgG antibody	Positive
Herpes simplex 2 IgG antibody	Non-reactive
Alpha 1 antitrypsin level	Normal
Serum ceruloplasmin level	Normal
Smooth muscle antibody (SMA)	Negative
Anti-mitochondrial antibody (AMA)	Negative
Hepatitis A IgM antibody (HAV IgM)	Non-reactive

**Figure 2:**
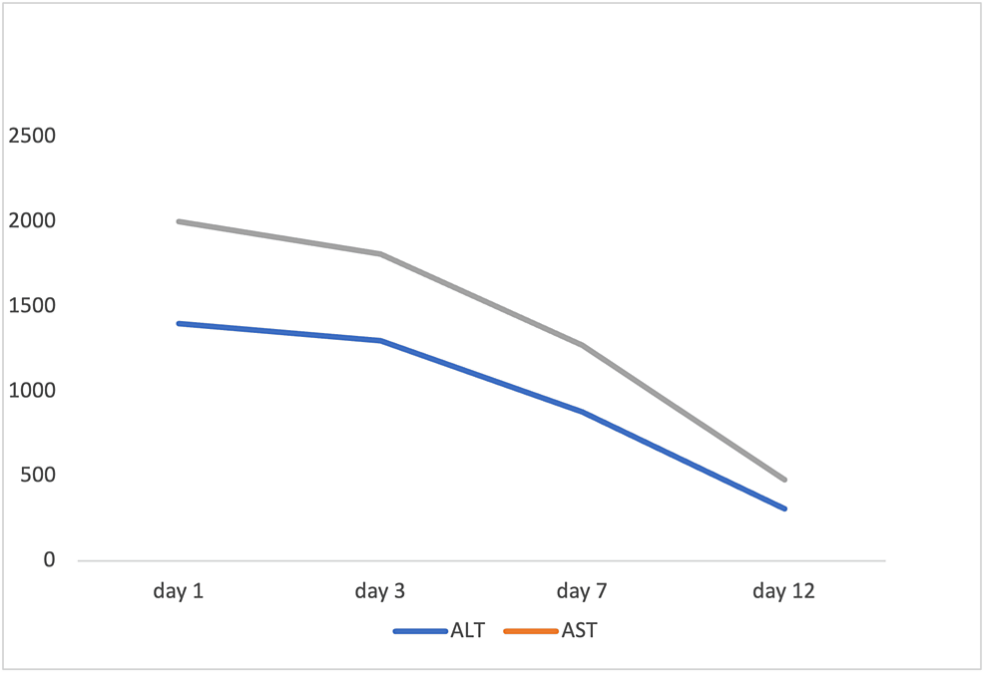
Graph showing the trend of liver function test All values are in U/L. AST: Aspartate Aminotransferase; ALT: Alanine Aminotransferase

During hospitalization the patient improved both clinically and in terms of liver function after inpatient treatment, leading to the decision to defer a liver biopsy. The trend of his liver function test is shown in Figure 2. The patient was discharged and scheduled for a follow-up with the outpatient gastroenterology clinic. The oral antiviral treatment for hepatitis B was continued as per the recommendation of the gastroenterologist. He was also advised to monitor his liver function closely to assess the response to the treatment and ensure the stabilization of his liver enzymes and bilirubin levels.

## Discussion

The presented case of a 30-year-old male with abdominal pain, jaundice, and a history of acetaminophen use raises several diagnostic challenges. The patient's initial presentation with severe abdominal pain, elevated liver function tests, and jaundice led to the suspicion of drug-induced hepatitis, particularly due to acetaminophen overdose. Acetaminophen toxicity is a well-recognized cause of acute liver injury, marked by specific clinical and laboratory features. These include elevated transaminases and increased bilirubin levels. Additionally, patients may exhibit symptoms such as nausea, vomiting, abdominal pain, and jaundice, while laboratory findings might also reveal prolonged prothrombin time and an elevated international normalized ratio [4]. NAC, an antidote for acetaminophen overdose, was promptly administered to this patient [5]. This decision was made without waiting for the patient's acetaminophen levels due to the worsening condition at presentation. The gastrointestinal team recommended NAC intervention, recognizing the imminent risk of the patient progressing into liver failure. This underscores the critical importance of early intervention in such cases.

However, the presence of a positive HBsAg introduced an unexpected twist in the diagnosis. Concurrent hepatitis B infection is an important consideration in patients with acute hepatitis, as it can exacerbate liver injury and complicate the clinical course. While this patient had a positive HBsAg, the hepatitis B e antigen was non-reactive, and the viral load was relatively low (3.32 log IU/mL). These findings raised questions about whether the acute hepatitis was solely due to hepatitis B or if there was a combination of drug-induced and viral hepatitis. The patient was initiated on antiviral treatment for hepatitis B, guided by the hepatologist's recommendations [6].

In the process of differential diagnosis, several other causes of hepatitis were also considered and subsequently ruled out. These included autoimmune hepatitis, assessed with markers such as anti-smooth muscle antibody and anti-mitochondrial antibody. The serological markers for hepatitis A, C, D, and E were all non-reactive, further narrowing down the potential etiologies. In the differential diagnosis, the possibility of cocaine-induced hepatitis was also considered. Chronic cocaine use has been associated with hepatotoxicity, with features such as elevated liver enzymes and histological changes [7]. However, establishing a direct causal link between cocaine and hepatitis can be challenging, as multiple factors, including concomitant substance abuse, can contribute to liver injury [8]. Nevertheless, given the complexity of this case, the exact contribution of cocaine to the hepatic pathology remains unclear and requires further investigation.

The discrepancy between the CT scan findings of a prominent IVC and the negative findings on ECHO raised an interesting point [9]. While the prominent IVC could have been associated with right heart congestion, it did not correspond with the absence of findings related to RHF or valvular pathology on ECHO. This highlights the need for comprehensive assessment and consideration of all clinical and radiological findings in complex cases, as they may not always align neatly with the working diagnosis.

The patient showed clinical improvement and enhanced liver function following inpatient treatment in this clinical case. Consequently, the decision was made to postpone a liver biopsy due to its invasive nature and the perceived lack of immediate necessity. While this choice of not doing a biopsy had benefits, such as minimizing patient risk and reducing costs, it also had limitations, including the absence of a definitive diagnosis and missed opportunities for detailed information and tailored treatment planning. However, it highlights that refraining from a biopsy can be justified in certain circumstances.

The management of this patient involved a multidisciplinary approach, including medical intervention for hepatitis and substance use counseling. The patient's response to treatment and commitment to behavioral change were positive indicators of a favorable outcome. In conclusion, this case emphasizes the complexity and challenges in diagnosing patients with acute hepatitis. It underscores the importance of a thorough evaluation, considering multiple potential etiologies, and highlights the need for ongoing monitoring and follow-up to ensure optimal patient care.

## Conclusions

In conclusion, this case report underscores the complex diagnostic challenges faced by patients with acute hepatitis. Initial suspicions of drug-induced hepatitis, notably due to acetaminophen overdose, can be further complicated by unexpected findings, as demonstrated in this case with the concurrent presence of hepatitis B infection. It is worth noting that this case is likely multifaceted, involving a combination of acetaminophen-induced drug injury, viral hepatitis, and potentially some element of cocaine-induced liver injury. The intricacies of this case emphasize the importance of a comprehensive and nuanced diagnostic approach in managing patients with acute hepatitis. The comprehensive, multidisciplinary approach to this patient's care, which involved both medical interventions for hepatitis and substance use counseling, led to positive clinical outcomes. The case underscores the significance of thorough evaluation, consideration of multiple potential etiologies, and the need for continuous monitoring and follow-up to ensure optimal patient care in cases of acute hepatitis. It also emphasizes the complexity of establishing causality in cases involving multiple contributing factors, such as cocaine use, and the importance of carefully weighing diagnostic invasiveness with patient safety and cost-effectiveness.

## References

[1] Lee WM (2017). Acetaminophen (APAP) hepatotoxicity - isn’t it time for Apap to go away?. J Hepatol.

[2] Nourjah P, Ahmad SR, Karwoski C, Willy M (2006). Estimates of acetaminophen (paracetomal)-associated overdoses in the United States. Pharmacoepidemiol Drug Saf.

[3] Fontana RJ (2008). Acute liver failure including acetaminophen overdose. Med Clin North Am.

[4] Yoon E, Babar A, Choudhary M, Kutner M, Pyrsopoulos N (2016). Acetaminophen-induced hepatotoxicity: a comprehensive update. J Clin Transl Hepatol.

[5] Heard KJ (2008). Acetylcysteine for acetaminophen poisoning. N Engl J Med.

[6] Morelli G, Perrella A, Sbreglia C, Bellopede P, Riccio V, Perrella O (2009). Antiviral therapy in acute viral hepatitis B: why and when. Infect Agent Cancer.

[7] Mitchell MC, Rogers C (2023). A case of cocaine-induced acute liver failure reversed with N-acetylcysteine. Cureus.

[8] Dolkar T, Hamad AM, Han MM, Thu MB, Gayam VR (2022). Cocaine and opioid-induced acute liver injury: a rare case report. Cureus.

[9] Malik A, Chaudhry M, Abdullah M, Inam SH, Iqbal N (2020). Idiopathic dilatation of inferior vena cava: a case report. Cureus.

